# Comparison of the Burrowing Ability of Different Groups of Manila Clams (*Ruditapes philippinarum*)

**DOI:** 10.3390/biology14060689

**Published:** 2025-06-12

**Authors:** Xiang Li, Jianing Wang, Zelin Zhang, Jin Wen, Yu Li, Haoyang Zhang, Pan Lu, Lei Chen

**Affiliations:** 1 College of Fisheries and Life Sciences, Dalian Ocean University, Dalian 116023, China; 15141432573@163.com (X.L.); 17866560812@163.com (Z.Z.); wenjindlou@163.com (J.W.); 19841146878@163.com (Y.L.); 2 College of Marine Science and Environment, Dalian Ocean University, Dalian 116023, China; 13624162670@163.com (J.W.); 13940850232@163.com (H.Z.); 15734144698@163.com (P.L.); 3 Center for Marine Ranching Engineering Science Research of Liaoning Province, Dalian Ocean University, Dalian 116023, China

**Keywords:** *Ruditapes philippinarum*, group, burrowing rate, particle size of substrate, sowing mode

## Abstract

Clams from the Fujian group, the Laizhou group, and the zebra strain group were used in this investigation; their shell lengths were 1.0 cm, 1.5 cm, and 2.0 cm, respectively. Tests were conducted on substrates with particle sizes ranging from 151 to 180 µm, 181 to 250 µm, 251 to 425 µm, and 426 to 850 µm. Both centralized and decentralized sowing modes were used. According to the findings, the clams with the 1.0 cm shell length had the highest burrowing rate and the lowest ET_50_. From 0 to 120 min, the burrowing rate of the zebra strain group was higher than that of the other groups. Clams with shell lengths of 1.0 cm and 1.5 cm had similar burrowing rates at the end of the test, with the zebra strain group having the highest burrowing rate. Manila clams burrowed more quickly when the substrate’s particle sizes were between 181 and 425 µm. The clam burrowing rates in the decentralized sowing mode were high during the first 20 min of the test, but at the end of the test, there was no significant difference between the two sowing modes (*p* > 0.05). In summary, there were differences in the burrowing ability among the three groups. The clams with a shell length of 1.0 cm in the three groups had a higher burrowing efficiency, and the decentralized sowing mode was more conducive to the clams quickly burrowing into the substrate.

## Simple Summary

The Manila clam (*Ruditapes philippinarum*) is the world’s second most important bivalve mollusk to be commercially farmed, and the Manila clam is a species that lives in the substrate. The burrowing ability is of great significance for clams to protect themselves from predators and water currents. At present, the clam groups involved in aquaculture in the northern sea of China include the Fujian group, the Laizhou group, and the zebra strain group. In this study, we investigated the burrowing ability of different Manila clam groups for the first time by comparing the burrowing ability of varying sizes within the Fujian, Laizhou, and zebra strain groups across different substrate sizes. The impact of the sowing mode on their burrowing ability was also explored. The results indicated that the burrowing ability of different groups and clam sizes varied, with the zebra strain group having a relatively stronger burrowing ability. In addition, the particle size of the substrate and the sowing mode also had an impact on the burrowing efficiency of clams from the three groups. This work will be of great significance for the selection of seedling groups in the aquaculture of clams.

## 1. Introduction

The Manila clam (*Ruditapes philippinarum*), a marine bivalve widely distributed across the Pacific and Indian Ocean coastlines [1], inhabits both northern and southern Chinese coastal waters and ranks among China’s four major cultured shellfish species [2]. The ability of shellfish to burrow is extremely critical for their survival and growth, which can help them to withstand the impact of currents and escape predation by natural enemies [3,4]. A considerable amount of research has been conducted on bivalves’ burrowing habits, primarily concentrating on behavioral ecology, environmental adaptation, and physiological causes. The burrowing behavior of the estuarine clam (*Rangia cuneata*) [5], razor clam (*Sinonovacula constricta*) [6], ark shell (*Scapharca broughtonii*) [7], and Manila clam (*R. philippinarum*) [8,9] has been reported. Clams’ burrowing behavior is a complicated process that is influenced by a number of variables, including the type of substrate and the water quality environment, including the salinity, temperature, and substrate particle size [10,11,12,13,14]. Only ark shells larger than 6 mm have the capacity to burrow, according to Kim et al. [7]. In the investigation of *Mactra veneriformis*, Nakamura et al. discovered that its burrowing rates were 30%, 90%, and 100% at salinities of 6.7, 11.2, and 25 [15]. According to Sakurai et al., *Ruditapes philippinarum*’s burrowing speed increased from 15 °C to 25 °C but dropped from 25 °C to 35 °C, accompanied by an increase in aberrant burrowing behavior [16]. The burrowing efficiency serves as a key viability indicator, where higher efficiency correlates with enhanced survival prospects [17]. Due to the differences in the shell shape and tolerance among different groups of clams, their burrowing habits vary [18].

However, single geographic groups have been the main focus of previous research. There are still few comparative studies on the burrowing habits of clam groups in various geographic locations. Nowadays, coastal mariculture of the Manila clam in the north and south primarily cultivates the Fujian group. This group does, however, have a comparatively low tolerance for cold. The Bohai Sea’s Laizhou Bay is home to the Laizhou group by nature. These clams’ very thin shells are frequently light red, giving them the local nickname “Laizhou Red”. The zebra strain group is a new kind that was created by the Dalian Ocean University team led by Professor Yan Xiwu, and it is distinguished by its high stress endurance and lovely patterns. These three groups are currently the main clam farming groups in northern China.

In this test, different substrate conditions were set indoors to observe the burrowing behaviors of clams from the Fujian group, the Laizhou group and the zebra strain group, and this test compared the effects of the clam size and sowing mode on the burrowing efficiency of clams. The aim was to investigate the burrowing ability of clams from different groups and to determine the suitable substrate conditions for them. This research will provide important references for selecting suitable groups of clams for aquaculture in marine areas.

## 2. Materials and Methods

### 2.1. Materials

**Figure 1:**
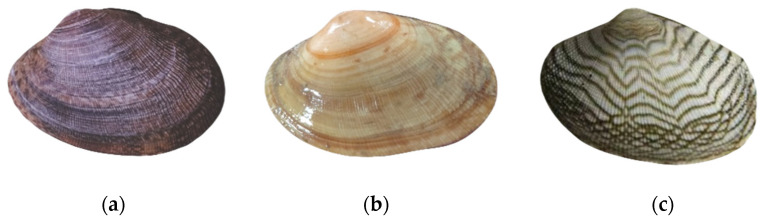
Illustration of the three clam groups. (**a**) Clam from the Fujian group. (**b**) Clam from the Laizhou group. (**c**) Clam from the zebra strain group.

Three groups of clams were selected for this test: the Fujian group (FJ) was taken from the offshore seabed area of Dandong, Liaoning China; the Laizhou group (LZ) was taken from the shallows area of Laizhou, Shandong China; and the zebra strain group (ZS) was taken from an intermediate breeding pond located in Panjin Liaoning China (Figure 1). The clams used in the test were all healthy individuals with undamaged shells, and they were divided into the 1.0 cm, 1.5 cm and 2.0 cm shell length groups. The clams were temporarily reared for 7 d in a glass tank (Dalian Huixin Titanium Equipment Development Co., Ltd., Dalian, Liaoning, China) at the Key Laboratory of Mariculture and Stock Enhancement in North China’s Sea, Ministry of Agriculture and Rural Affairs, Dalian Ocean University, with a water temperature of 15 ± 1 °C and a salinity of 30 ± 1. They were continuously aerated for 24 h. They were fed once a day with *Chlorella vulgaris* and spirulina powder (Fuqing Xindaze Spirulina Co., Ltd., Fuzhou, Fujian, China). The seawater used was taken from the Heishijiao sea area in Dalian, and it was used after sedimentation and sand filtration. The water was changed once a day before feeding, and the amount of water changed was 50%.

**Figure 2:**
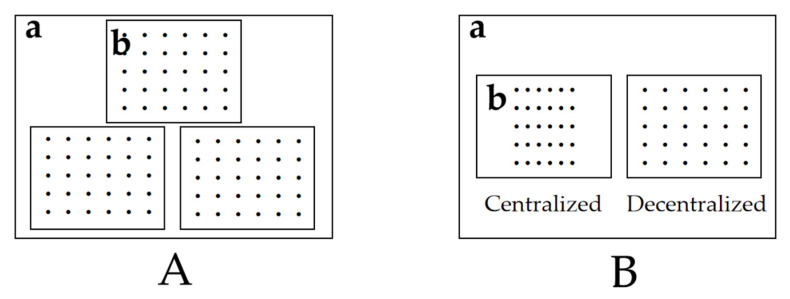
(**A**): Tests of the ET_50_ and burrowing rate of the three groups of clams. (**B**): Tests of the centralized and decentralized sowing modes. Note: (a) Large sink; and (b) small sink. “.” represents the clam.

The substrate used in the test was taken from the intertidal zone of the Dalian Heishijiao marine area, and its particle size was sieved by a sampling sieve (Hebei Anping Zhenxing Sieve Factory, 5–100 mesh), soaked in 0.5% solution of sodium hypochlorite (Dandong Quanyou Technology Co., Ltd., Dandong, Liaoning, China) for 24 h, rinsed with water, and then loaded into small plastic tanks (30 cm × 21 cm × 12 cm, Jiangsu Lishen Plastic Industry Technology Co., Ltd., Wuxi, Jiangsu, China) with a thickness of 7 cm. The small plastic tank with the substrate was placed at the bottom of the large plastic tank (65 cm × 50 cm × 48 cm, Jiangsu Lishen Plastic Industry Technology Co., Ltd., Wuxi, Jiangsu, China), and the large plastic tank was filled with seawater (Figure 2).

### 2.2. Test Method

Vertical shell time:

Thirty clams of the same size were taken and placed in a small plastic tank, and the time taken for 50% of the clams to erect their shells (ET_50_) was recorded from the time the clams landed on the surface of the substrate. Three replicates were set up for each group. No water change was allowed during the test period and the aeration was continuous (Figure 2A).

Before the test, the shell length, shell width and shell height of the clams were measured using vernier calipers, and the body mass was weighed using an electronic balance.

**Figure 3:**
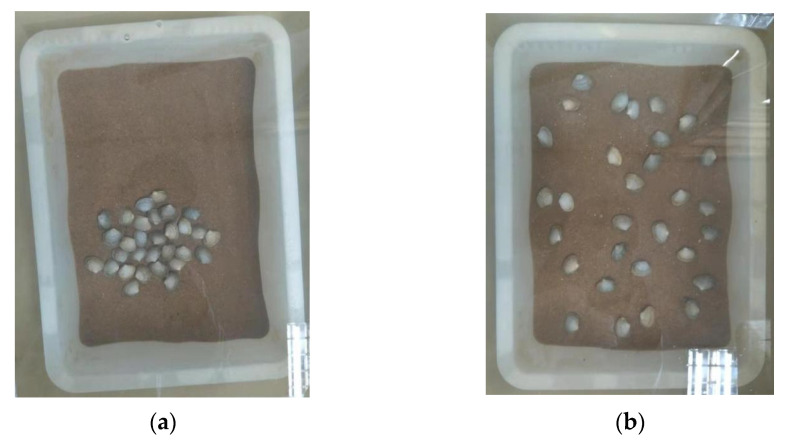
Centralized and decentralized sowing of Manila clams. (**a**) Centralized sowing. (**b**) Decentralized sowing.

Each group included clams of three sizes: shell length 1.0 cm, 1.5 cm, and 2.0 cm. (Table 1). In addition, four sizes of substrate particle were considered: 151–180 µm, 181–250 µm, 251–425 µm, and 426–850 µm. Finally, two modes of sowing were adopted: centralized sowing and decentralized sowing. The former referred to placing the clams in the central area of the substrate surface, and the latter referred to evenly placing the clams on the surface of the substrate (Figure 2B and Figure 3).

Burrowing rate:

Thirty clams of the same specification were taken and put into a small plastic tank. The clams were observed from the time they landed on the surface of the substrate. The frequency of observation was once every 10 min for the first hour and once every hour thereafter. The number of clams burrowing into the substrate was recorded after 24 h. Each observation time was limited to 1 min. Three replicates were set up for each group. Continuous aeration was provided for 24 h without a water change.

Calculation formula: Burrowing rate = (number of clams burrowing into the sand after 24 h/total number of clams tested) × 100%.

Note: The criterion was that the clams were completely burrowed into the substrate.

### 2.3. Data Processing

Microsoft Office 2021 software was used to collate the experimental data, which are presented as the mean ± standard deviation (SD); meanwhile, analysis of variance (ANOVA) and Duncan multiple comparisons were performed using SPSS 22.0 software. The significance level was set at 0.05.

## 3. Results

### 3.1. Effects of Substrate Conditions on ET_50_ of Clams from Different Groups

**Figure 4:**
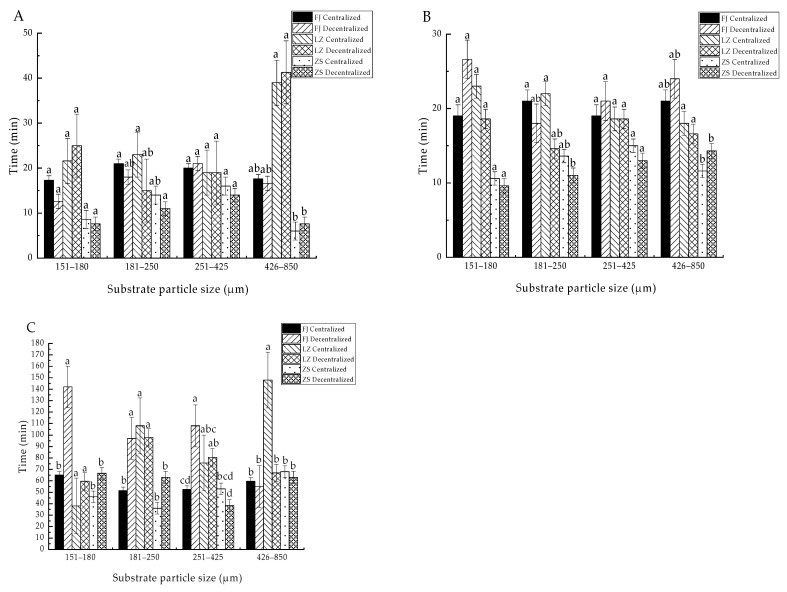
The ET_50_ of clams from three groups under four types of substrate conditions. Note: (**A**) Clams with a 1.0 cm shell length; (**B**) clams with a 1.5 cm shell length; and (**C**) clams with a 2.0 cm shell length. Under the same substrate conditions, different letters indicate significant differences (*p* < 0.05) and the same letters indicate no significant differences (*p* < 0.05), et sequentia. FJ Centralized: Manila clams of Fujian group using centralized sowing mode, FJ Decentralized: Manila clams of Fujian group using decentralized sowing mode, LZ Centralized: Manila clams of Laizhou group using centralized sowing mode, LZ Decentralized: Manila clams of Laizhou group using decentralized sowing mode, ZS Centralized: Manila clams of zebra strain group using centralized sowing mode, ZS Decentralized: Manila clams of zebra strain group using decentralized sowing mode, et sequentia.

The ET_50_ of the clams with a shell length of 1.0 cm ranged from 8 to 24 min under the substrate conditions of particle size 151–180 µm, and there was no significant difference between the ET_50s_ of the three groups and the two sowing modes (*p* > 0.05). Under the substrate conditions of 181–250 µm, the ET_50_ ranged from 11 to 22 min, and there was only a significant difference in the ET_50_ between the zebra strain group of decentralized sowing and the other two groups of centralized sowing (*p* < 0.05). Under the substrate conditions of 251–425 µm, the ET_50_ ranged from 13 to 21 min, and there was no significant difference between the ET_50s_ of the three groups and the two sowing modes (*p* > 0.05). Under the substrate conditions of 426–850 µm, the ET_50_ ranged from 6 to 42 min, and there was no significant effect on the ET_50_ of the three groups with the two sowing modes (*p* > 0.05), but the ET_50_ of the Laizhou group was significantly higher than that of the zebra strain group (*p* < 0.05) (Figure 4A).

The ET_50_ of the clams with a shell length of 1.5 cm ranged from 10 to 27 min under the substrate conditions of particle size 151–180 µm, and there were no significant differences in the ET_50_ of the three groups and the two sowing modes (*p* > 0.05). Under the substrate conditions of 181–250 µm, the ET_50_ ranged from 11 to 22 min, and there was only a significant difference in the ET_50_ between the zebra strain group of decentralized sowing and the other groups of centralized sowing (*p* < 0.05). Under the substrate conditions of 251–425 µm and 426–850 µm, the ET_50_ ranged from 13 to 21 min and from 12 to 24 min, respectively, and there were no significant differences in the ET_50_ for any of the three groups and for the two sowing modes (*p* > 0.05) (Figure 4B).

The ET_50_ of the clams with a shell length of 2.0 cm ranged from 35 to 140 min under the substrate condition of particle size 151–180 µm, and the ET_50_ of the Fujian group in the decentralized sowing mode was significantly higher than that of the other groups (*p* < 0.05). Under the substrate conditions of 181–250 µm, the ET_50_ ranged from 36 to 103 min. The sowing mode had a significant effect on the Fujian group (*p* < 0.05), but no significant differences were observed in the ET_50_ of the Laizhou group and the zebra strain group (*p* > 0.05). The ET_50_ of the Fujian group in the decentralized sowing and the Laizhou group in the two sowing modes was significantly higher than that of the Fujian group in the centralized sowing and the zebra strain group in the two sowing modes (*p* < 0.05). Under the substrate conditions of 251–425 µm, the ET_50_ ranged from 38 to 103 min, and there was no significant effect on the ET_50_ of the Laizhou group and the zebra strain group in the two sowing modes (*p* > 0.05), but there was a significant effect on the ET_50_ of the Fujian group (*p* < 0.05). The ET_50_ of the zebra strain group in the decentralized sowing was the lowest among all the groups, while the value of the Fujian group in the decentralized sowing was the highest. Under the substrate conditions of 426–850 µm, the ET_50_ ranged from 54 to 148 min, and the ET_50_ of the Laizhou group in the centralized sowing was significantly higher than that of the other groups (*p* < 0.05). There was no significant difference in the ET_50_ among the other groups (*p* > 0.05) (Figure 4C).

### 3.2. Effect of Substrate Conditions on Burrowing Rate of Clams from Different Groups

#### 3.2.1. Burrowing Rate of Clams with Shell Length of 1.0 cm

**Figure 5:**
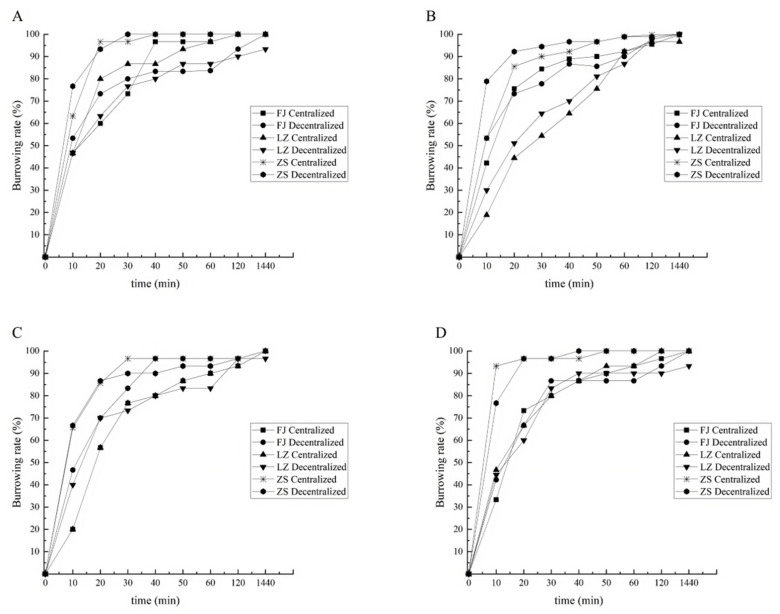
Burrowing rate of 1.0 cm clams in four types of substrate conditions. Note: (**A**) Substrate conditions of particle size 151–180 µm; (**B**) substrate conditions of particle size 181–250 µm; (**C**) substrate conditions of particle size 251–425 µm; and (**D**) substrate conditions of particle size 426–850 µm, et sequentia.

Under the substrate conditions of particle size 151–180 μm, the highest burrowing rate was found in the zebra strain group at 0–60 min, followed by the Fujian group, while the Laizhou group had the lowest burrowing rate. However, there was no significant difference among these groups (*p* > 0.05). For the Fujian and Laizhou groups, the different sowing modes had some effects on the burrowing rate of the clams at 0–60 min. Overall, from 0 to 10 min, the burrowing rate of the clams in the decentralized sowing mode was higher than that in the centralized sowing mode during the same period, but there was no significant difference (*p* > 0.05). In the later period, the burrowing rate of the clams in the centralized sowing mode was higher than that in the decentralized sowing mode for both the Fujian and Laizhou groups. Meanwhile, there was no significant difference in the burrowing rate of the zebra strain group between the two sowing modes (Figure 5A).

Under the substrate conditions of particle size 181–250 µm, there was a significant difference in the burrowing rates between the Laizhou group and the zebra strain group at 0–40 min (*p* < 0.05). At 10 min, the burrowing rate of the Laizhou group was only 24.45%, compared to 66.11% for the zebra strain group. At 20 min, the burrowing rate of the Fujian group was 74.44%, compared to 47.78% for the Laizhou group. From 0 to 40 min, the burrowing rates of the Fujian and zebra strain groups were significantly higher than that of the Laizhou group (*p* < 0.05). At 60 min, there was no significant difference in the burrowing rates of the three groups (*p* > 0.05). From 0 to 10 min, the sowing modes had a significant effect on the burrowing rate of the zebra strain group (*p* < 0.05), with the burrowing rate was significantly higher for the decentralized sowing mode than for the centralized sowing mode. The burrowing rate of the clams using the centralized sowing mode increased rapidly from 10 to 60 min. At 1440 min, there was no significant effect on the burrowing rate of the three groups between the two sowing modes (*p* > 0.05) (Figure 5B).

Under the substrate conditions of particle size 251–425 µm, at 10 min, the mean burrowing rate of the Fujian and Laizhou groups was only 31.67%, while that of the zebra strain group was 66.16%. At 20 min, the mean burrowing rate of the Fujian and Laizhou groups was only 63.34%, while that of the zebra strain group increased to 86.12%. From 0 to 20 min, the burrowing rate of the zebra strain group was significantly higher than that of the Fujian and Laizhou groups (*p* < 0.05). From 50 to 1440 min, the burrowing rates of all three groups were close to each other, with no significant differences (*p* > 0.05). At 10 min, there was a significant difference in the burrowing rates between the Fujian and Laizhou groups under the two sowing modes (*p* < 0.05), with the decentralized sowing mode resulting in higher rates than the centralized sowing mode. From 10 to 20 min, the burrowing rate increased rapidly in the Fujian and Laizhou groups using the centralized sowing mode. By 30 min, the burrowing rates of the two sowing modes were close to each other. After 120 min, there was no significant effect of the two sowing modes on the burrowing rates of the clams (*p* > 0.05) (Figure 5C).

Under the substrate conditions of particle size 426–850 µm, the mean burrowing rate of the Fujian and Laizhou groups of 10 min was 41.39%, while that of the zebra strain group was 85%. At 20 min, the mean burrowing rate of the Fujian and Laizhou groups was 66.67%, while that of the zebra strain group increased to 96.67%. From 0 to 20 min, the burrowing rate of the zebra strain group was significantly higher than that of the Fujian and Laizhou groups (*p* < 0.05). From 30 to 1440 min, the burrowing rates of the three groups were close to each other, with no significant differences (*p* > 0.05). From 0 to 1440 min, the two sowing modes generally had no significant effect on the burrowing rates of the three groups (*p* > 0.05) (Figure 5D).

Under different substrate conditions, for the clams with a shell length of 1.0 cm at the beginning of the test (0–120 min), the zebra strain group had the highest burrowing rate, followed by the Fujian group, and the Laizhou group had the lowest. In the initial stage, the two sowing modes had a significant effect on the burrowing rate of the clams, with higher rates observed in the decentralized sowing mode than in the centralized sowing mode (*p* < 0.05). After 1440 min, the burrowing rates of the two sowing modes were close to each other, with no significant differences (*p* > 0.05). Under the substrate conditions of particle size 251–425 μm, the differences in the burrowing rates among the three groups were smallest.

#### 3.2.2. Burrowing Rate of Clams with Shell Length of 1.5 cm

**Figure 6:**
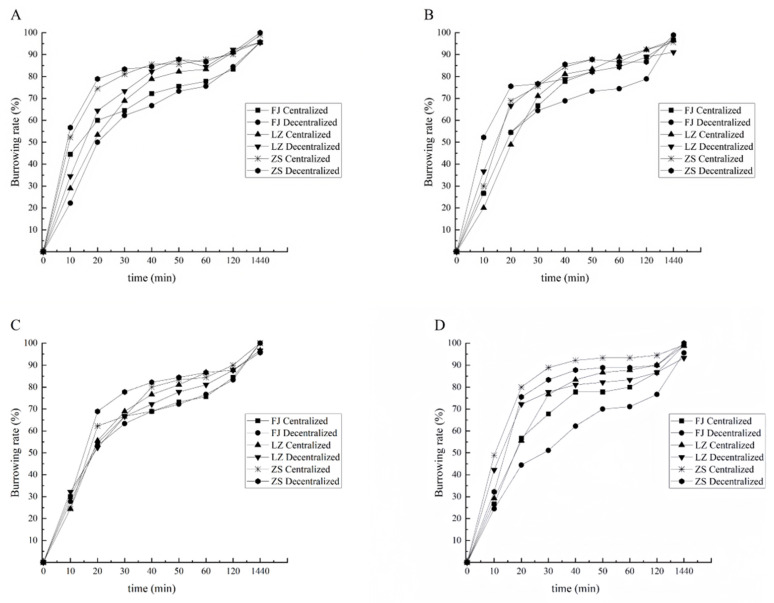
Burrowing rate of 1.5 cm clams in four types of substrate conditions.

Under the substrate conditions of particle size 151–180 μm, the highest burrowing rate was found in the zebra strain group at 0–120 min, followed by the Laizhou group, and the Fujian group had the lowest burrowing rate. Meanwhile, the burrowing rates of the Laizhou and zebra strain groups were higher with the decentralized sowing mode than with the centralized sowing mode. In contrast, the Fujian group had a higher burrowing rate with the centralized sowing mode than with the decentralized sowing mode. At 1440 min, there was no significant effect on the burrowing rates of the three groups between the two sowing modes (*p* > 0.05) (Figure 6A).

Under the substrate conditions of particle size 181–250 µm, the highest burrowing rate was found in the zebra strain group at 0–120 min, followed by the Laizhou group, while the Fujian group had the lowest burrowing rate. At 10 min, the burrowing rate of the zebra strain group using the decentralized sowing mode was significantly higher than that of the other groups (*p* < 0.05). Meanwhile, at 20 min, the burrowing rates of the other groups increased rapidly. From 0 to 30 min, the burrowing rate of the Laizhou group was higher with the decentralized sowing mode than with the centralized sowing mode. However, from 40 to 120 min, the burrowing rate of the Fujian group using the decentralized sowing mode was significantly lower than that of the other groups (*p* < 0.05). At 1440 min, there was no significant effect on the burrowing rates of the three groups between the two sowing modes (*p* > 0.05) (Figure 6B).

Under the substrate conditions of particle size 251–425 μm, the highest burrowing rate was found in the zebra strain group at 0–120 min, followed by the Laizhou group, while the Fujian group had the lowest burrowing rate. The burrowing rate of the zebra strain group was higher with the decentralized sowing mode than with the centralized sowing mode at 30 min (*p* < 0.05). However, for the rest of the time, there was no significant effect on the burrowing rates of the three groups between the two sowing modes (*p* > 0.05) (Figure 6C).

Under the substrate conditions of particle size 426–850 µm, the zebra strain group had the highest burrowing rate, followed by the Laizhou group, while the Fujian group had the lowest burrowing rate. From 20 to 120 min, the burrowing rates of the Laizhou and zebra strain groups were significantly higher than those of the Fujian group (*p* < 0.05). At 1440 min, there was no significant effect on the burrowing rates of the three groups between the two sowing modes (*p* > 0.05) (Figure 6D).

Under different substrate conditions, for the clams with a shell length of 1.5 cm, the highest burrowing rate was found in the zebra strain group from 0 to 120 min, followed by the Laizhou group, while the Fujian group had the lowest burrowing rate. The two sowing modes had some effects on the burrowing rates of the three groups, with the most significant effect observed in the Fujian group. Specifically, the burrowing rate of the Fujian group was higher with the centralized sowing mode than with the decentralized sowing mode. During the test, under the substrate conditions of particle size 251–425 μm, the differences in the burrowing rates among the three groups were the smallest.

#### 3.2.3. Burrowing Rate of Clams with Shell Length of 2.0 cm

**Figure 7:**
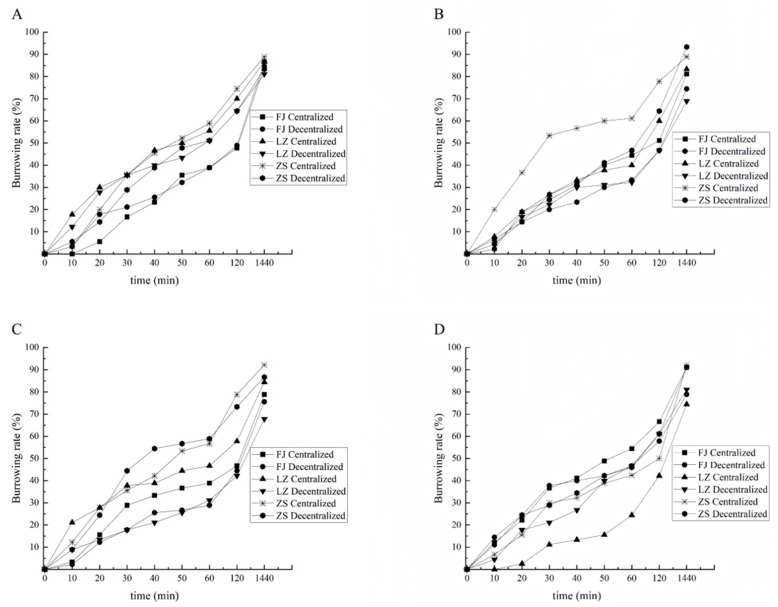
Burrowing rate of 2.0 cm clams in four types of substrate conditions.

Under the substrate conditions of particle size 151–180 µm, the burrowing rates of the Laizhou and zebra strain groups were higher than those of the Fujian group. Meanwhile, for the Fujian group, the burrowing rate with the decentralized sowing mode was higher than that with the centralized sowing mode from 0 to 30 min, but there was no significant difference between the two sowing modes after 40 min (*p* > 0.05). After 120 min, the burrowing rate of the Fujian group increased rapidly, and at 1440 min, the burrowing rates of all three groups were close to each other (*p* > 0.05) (Figure 7A).

Under the substrate conditions of particle size 181–250 µm, the burrowing rate of the zebra strain group was higher than that of the Fujian and Laizhou groups. Specifically, the burrowing rate of the zebra strain group using the centralized sowing mode was significantly higher than that of the other groups (*p* < 0.05), while the burrowing rates of the other groups showed no significant differences (*p* > 0.05). At 30 min, the burrowing rate of the zebra strain group using the centralized sowing mode reached 53.33%, while the burrowing rates of the other groups were all at least 15.55%. However, the difference in the burrowing rate between the two sowing modes was not significant for the Fujian and Laizhou groups (*p* > 0.05), but it was significant for the zebra strain group (*p* < 0.05) (Figure 7B).

Under the substrate conditions of particle size 251–425 µm, the zebra strain group had the highest burrowing rate. Meanwhile, the two sowing modes had some effects on the burrowing rates of the three groups. There was no significant effect of the two sowing modes on the burrowing rate of the zebra strain group (*p* > 0.05). For the Fujian and Laizhou groups, the burrowing rate of the clams using the centralized sowing mode was generally higher than that using the decentralized sowing mode during the same period (Figure 7C).

Under the substrate conditions of particle size 426–850 µm, the burrowing rate of the Laizhou group with the decentralized sowing mode was significantly higher than that with the centralized sowing mode (*p* < 0.05). Meanwhile, from 20 to 60 min, the burrowing rate of the Laizhou group using the centralized sowing mode was significantly lower than that of the other groups (*p* < 0.05). At 50 min, the burrowing rate of the Laizhou group with the centralized sowing mode was only 15.55%, while the rates of the other groups were all at least 36.66%. For the Fujian and zebra strain groups, there was no significant effect on burrowing rate between the two sowing modes (*p* > 0.05) (Figure 7D).

Under different substrate conditions, for the clams with a shell length of 2.0 cm, the zebra strain group had the highest burrowing rate from 0 to 120 min. Meanwhile, under large particle size (426–850 µm) substrate conditions, the Laizhou group had the lowest burrowing rate, especially with the centralized sowing mode. At the beginning of the test, the two sowing modes affected the burrowing rates of the three groups (*p* < 0.05).

## 4. Discussion

### 4.1. Comparison of Burrowing Ability of Different Groups of Clams

Using the ET_50_ and burrowing rate as the determination indices, the zebra strain group had the most outstanding burrowing ability among the clams of the same size. This phenomenon may be related to the characteristics of the zebra strain group, which not only has beautiful patterns but also possesses strong resilience, such as resistance to low salinity, low temperature, and high temperature [19,20]. This strong ability to adapt to the environment may be an important reason for its outstanding burrowing ability [21]. In terms of the shell length, Zhang et al. [22] found that the burrowing speed of *Paphia undulata* with shell lengths of 0.3, 0.5, and 1.0 cm increased with an increasing shell length. In this test, the clams used were shellfish larvae with shell lengths of 1.0 to 2.0 cm, and their burrowing speed showed a slowdown with an increasing shell length. The reason for this is that individuals with a shell length of 0.3–1.0 cm are in the early stages of shellfish larvae, and their locomotor abilities are relatively weak. In contrast, the clams used in this test had relatively more locomotor ability, and the larger the individual, the more energy and time it took to flip from a flat to an upright position. This result is similar to the findings of St-Onge et al. in softshell clam [23] and Abarca et al. in *Mulinia edulis* [24]. The burrowing ability of shellfish is affected by the individual size, locomotor ability and individual density [23,25]. As shellfish grow, the increase in body size may increase the resistance during burrowing, thus reducing the efficiency of burrowing [26].

### 4.2. Effect of the Substrate Conditions on the Burrowing Ability of Clams

The substrate is an important abiotic factor affecting benthic shellfish, influencing their geographic distribution, survival, growth, and behavior [27,28]. The ability of shellfish to burrow into substrates of different particle sizes varies, indicating that benthic shellfish are selective with respect to the substrate particle size. The burrowing time and burrowing rate can quantitatively reflect the adaptability of benthic shellfish to the substrate conditions [29,30].

In this test, the clams took the shortest time for burrowing under the substrate particle sizes of 181–250 µm and 251–425 µm, and the clams took a longer time for burrowing under the substrate particle sizes of 151–180 µm and 426–850 µm. The reason for this is that the clams rely on the axe foot to rub the substrate when burrowing, and the difference in the friction of the substrate affected the burrowing ability of the clams. In the appropriate particle size range, the friction was suitable, and the clams were able to use the friction between the axe foot and the substrate quickly to complete the action of erecting shell and digging. If the particle size is too coarse or too fine, the burrowing time will be prolonged [31]. When the particle size is too fine, the gap between the particles is smaller, resulting in a more compact substrate, and the compactness of the fine-grained substrate increases the resistance of the shellfish’s axe feet to digging, making the shellfish need to consume more energy and time to complete the burrowing. When the particle size is too coarse, the gap between the particles is larger, and the substrate structure is loose, so it is difficult for the shellfish’s axe feet to effectively grip the coarse particles when digging, resulting in a reduction in the efficiency of the burrowing and an increase in the burrowing time [32,33]. The burrowing rate of *Donax trunculus* L. with a shell length of 0.5–2.5 cm was higher under the condition of medium (250–500 µm) and coarse (500–1000 µm) substrate conditions [34]. Under conditions of too fine or too coarse substrate, the burrowing efficiency of *Donax trunculus* L. was relatively low, and *Ruditapes philippinarum* and *Donax trunculus* L. were similar.

### 4.3. The Effect of the Sowing Mode on the Burrowing Ability of Clams

In this test, the burrowing rates of the three clam groups were influenced by the two sowing modes and showed some regularity. At the beginning of the test, the clams that were dispersed using the decentralized sowing mode were able to quickly initiate locomotion and burrow due to the ample space available on the substrate. This behavior, which may be an instinctive response to adapt to the environment and reduce the risk of exposure when sufficient space is available, resulted in a higher burrowing rate. In contrast, the clams that were sown using the centralized mode faced space constraints. They had to rely on their foot to move and find suitable space for burrowing. Large-sized clams, in particular, had to spend a long time dispersing due to their limited body flexibility. They could only start burrowing after obtaining appropriate space conditions, which led to a lower burrowing rate at the beginning of the test. At 1440 min, the two sowing modes had no significant effect on the burrowing rate of the three clam groups. This may be related to the compensatory effect of clustering behavior, which is different from the burrowing pattern of the ark shell (*Scapharca subcrenata*). In the test of the burrowing behavior of the ark shell [35] under different sowing modes, the ark shell had the characteristics of clustering burrowing. In the experiment investigating the impact of the sowing modes on the burrowing of ark shell, it was observed that these clams exhibited a tendency to gather together before burrowing into the sand.

Significant morphological differences have driven the distinct burrowing survival strategies among different bivalve species [36]. The relatively broad and thick shell structure of the ark shell limits its locomotor capacity. Gregarious burrowing behavior helps loosen the substrate, thereby enhancing the overall burrowing efficiency [7]. This explains why they have developed a gregarious burrowing strategy to compensate for their limited individual mobility. In contrast, clams possess more compressed shells, conferring greater body flexibility and stronger locomotor ability. While clams optimize their individual burrowing ability to cope with environmental pressures, ark shell rely more heavily on collective coordination to achieve effective burrowing.

## 5. Conclusions

This study explored the variations in the burrowing ability of three clam groups by establishing the sizes of the clams, particle sizes of the substrate, and sowing modes. Analyses revealed that individuals with a shell length of 1.0 cm exhibited the fastest burrowing rate, while those measuring 2.0 cm burrowed relatively slower. The initial burrowing efficiency of the zebra strain clams was higher. The three groups of clams had the highest burrowing efficiency under sediment conditions with a particle size of 181–425 μm. Although the decentralized sowing mode did not affect the final burrowing rate, it markedly shortened the preparatory time, challenging the conventional understanding within sowing mode research. Notably, the clam groups demonstrated exceptional burrowing performance.

In the mariculture practice of clams, selecting an appropriate clam group and particle size of the substrate, combined with employing the decentralized sowing mode, can significantly reduce the preparatory time before burrowing. This facilitates rapid burrowing into the substrate, thereby reducing exposure to predators and washing away by water currents. Furthermore, developing eco-friendly artificial substrates based on the optimal particle size range will optimize the habitat for mariculture. These findings not only provide novel insights into the behavioral ecology of shellfish but also serve as a crucial reference for selecting seed groups of mariculture.

## Figures and Tables

**Table 1:** Specifications of the clams used in the burrowing tests.

Group	Size/cm	Shell Length/mm	Shell Width/mm	Shell Height/mm	Wet Weight/g
Fujian group	1.0	10.16 ± 0.66	3.97 ± 0.34	7.10 ± 0.53	0.17 ± 0.04
	1.5	14.80 ± 0.58	5.89 ± 0.57	10.50 ± 0.78	0.50 ± 0.11
	2.0	20.38 ± 0.88	9.14 ± 0.68	14.01 ± 0.94	1.69 ± 0.28
Laizhou group	1.0	9.94 ± 0.68	3.75 ± 0.23	6.80 ± 0.49	0.18 ± 0.03
	1.5	14.87 ± 0.42	5.96 ± 0.35	10.34 ± 0.49	0.59 ± 0.08
	2.0	20.08 ± 0.86	8.34 ± 0.66	13.49 ± 0.79	1.56 ± 0.25
Zebra strain group	1.0	10.26 ± 0.60	4.22 ± 0.30	7.11 ± 0.38	0.19 ± 003
	1.5	15.05 ± 0.87	5.95 ± 0.47	10.19 ± 0.54	0.61 ± 0.11
	2.0	20.16 ± 0.95	9.35 ± 0.61	15.07 ± 0.80	1.93 ± 0.27

## Data Availability

Date will be made available on request.
